# The de novo methylation activity of Dnmt3a is distinctly different than that of Dnmt1

**DOI:** 10.1186/1471-2091-6-6

**Published:** 2005-03-30

**Authors:** Chih-Lin Hsieh

**Affiliations:** 1 Department of Urology and Department of Biochemistry and Molecular Biology, University of Southern California, 1441 Eastlake Ave., Rm 5420, Norris Cancer Center, Los Angeles, CA 90033, USA

## Abstract

**Background:**

Though Dnmt1 is considered the primary maintenance methyltransferase and Dnmt3a and Dnmt3b are considered de novo methyltransferases in mammals, these three enzymes may work together in maintaining as well as establishing DNA methylation patterns. It has been proposed that Dnmt1 may carry out de novo methylation at sites in the genome with transient single-stranded regions, such as replication origins, and then spread methylation from these nucleation sites in vivo, even though such activity has not been reported.

**Results:**

In this study, we show that Dnmt3a does not act on single-stranded substrates in vitro, indicating that Dnmt3a is not likely to initiate DNA methylation at such proposed nucleation sites. Dnmt3a shows similar methylation activity on unmethylated and hemimethylated duplex DNA, though with some substrate preference. Unlike Dnmt1, pre-existing cytosine methylation at CpG sites or non-CpG sites does not stimulate Dnmt3a activity in vitro and in vivo.

**Conclusion:**

The fact that Dnmt3a does not act on single stranded DNA and is not stimulated by pre-existing cytosine methylation indicates that the de novo methylation activity of Dnmt3a is quite different from that of Dnmt1. These findings are consistent with a model in which Dnmt3a initiates methylation on one of the DNA strands of duplex DNA, and these hemimethylated sites then stimulate Dnmt1 activity for further methylation.

## Background

DNA cytosine-5-methyltransferases (Dnmts) catalyze the methyl transfer from S-adenosyl-L-methionine to the cytosine in CpG dinucleotides. Four active DNA methyltransferases, Dnmt1, Dnmt2, Dnmt3a, and Dnmt3b, have been reported in mammals. These four enzymes contain highly conserved DNA methyltransferase motifs; however, genetic and biochemical studies have suggested functional differences between them. Dnmt1 is considered a maintenance DNA methyltransferase, though some de novo methylation activity has been described in vitro [1]. Dnmt3a and Dnmt3b are generally regarded as de novo DNA methyltransferases, even though it has been proposed that these enzymes may play a role in the maintenance of methylation in ES cells [2]. While the biological role of Dnmt2 remains unknown, it has been reported to methylate DNA at a very low level, and it may be involved in non-CpG methylation [3-6].

De novo methylation mostly occurs during embryonic development and plays an important role in genomic imprinting, X-inactivation, and transposon suppression in mammals. De novo methylation has been described to occur frequently in cancer [for a review, see [7]] and at sites of integrated exogenous DNA [8]. It has been proposed that de novo methyltransferases may participate in the maintenance of methylation based on the findings that Dnmt3a and/or Dnmt3b may play roles in restoring methylation at sites missed by Dnmt1 during replication in ES cells [2]. A Dnmt1-deficient cancer cell line showed little loss of DNA methylation, suggesting that the de novo methyltransferases can maintain a high level of DNA methylation in the absence of Dnmt1 in neoplastic cells [9]. A more dramatic loss of DNA methylation was observed when Dnmt1 and Dnmt3b were both absent from that cell line, suggesting the possible cooperation between these two enzymes [10]. Another study using antisense and RNAi strategies suggested that Dnmt1 is responsible for global and CpG island methylation in cancer cells [11]. These studies raise the possibility that Dnmt1 and the two de novo methyltransferases may work together in maintaining, as well as establishing, DNA methylation patterns.

Dnmt3a has been shown to have biochemical characteristics different than Dnmt1. Dnmt3a shows no preference for hemimethylated over fully unmethylated DNA substrates, whereas Dnmt1 has a strong preference for hemimethylated DNA [12,13]. Dnmt1 has no known preference other than CpG sites in vitro [14], whereas Dnmt3a appears to prefer CpG sites flanked by pyrimidines in vitro [15] and shows less specificity for CpG sites than Dnmt1 both in vitro and in vivo [13,16]. Dnmt3a generates asymmetrical methylation pattern on two DNA strands in vitro [15]. These differences reflect the methylation maintenance function of Dnmt1 and de novo methylation function of Dnmt3a.

It has been reported that pre-existing cytosine methylation at CpG as well as at non-CpG sites can stimulate de novo methylation activity of native and recombinant Dnmt1 on single-stranded oligonucleotides in vitro [1,17]. It has been hypothesized that single-stranded DNA formed during replication or repair might serve as a nucleation site for de novo methylation by Dnmt1 [17]. Purified murine Dnmt1 overexpressed in *E. coli *also showed increased de novo methylation activity when pre-existing cytosine methylation was present on double-stranded oligonucleotide substrates [18]. In another study, introduction of random CpG methylation into plasmid DNA stimulated methylation activity of DNA methyltransferases partially-purified from human placenta and murine liver [19]. A more recent study showed that fully methylated oligonucleotides can also stimulate Dnmt1 de novo methylation activity in trans [20]. These studies demonstrate that pre-existing cytosine methylation on oligonucleotides as well as on plasmids can stimulate the de novo methylation activity of Dnmt1 in vitro, even though this activity has not been confirmed in vivo. This stimulation may be due to the allosteric activation of the catalytic domain by the binding of Dnmt1 to methylated DNA [20]. In our previous studies [21-23], de novo methylation of the episome by endogenous Dnmt1 has not been detected in human cells regardless of the methylation status of the episome (CpG methylation at HhaI or HpaII sites, or non-CpG methylation at Dcm sites of the plasmid).

It is clear that Dnmt3a is not stimulated by pre-existing cytosine methylation at CpG sites on only one of the DNA strands because it does not prefer hemimethylated DNA over fully unmethylated DNA. While Dnmt3a does not appear to be stimulated by methylation at HpaII sites on a 310 bp DNA fragment [24], extensive testing has not been carried out. One study showed stimulation of Dnmt3a by hemimethylated CCG and fully methylated CWG; no stimulation by fully methylated CCG was detected [25]. It is curious that Dnmt3a is not stimulated by hemimethylated CpG, fully methylated CpG, and fully methylated CCG, but it is stimulated by hemimethylated CCG and fully methylated CWG in vitro [25]. In this study, we carried out experiments to determine whether Dnmt3a has similar de novo methylation activity on single-stranded substrates and whether it can be stimulated by pre-existing cytosine methylation, as is the case for Dnmt1.

## Results

### Dnmt3a has substrate preferences and does not act on single-stranded DNA substrates

**Figure 1:**
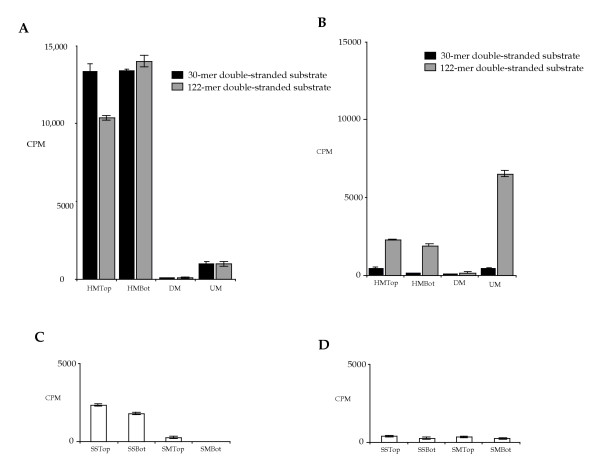
***In vitro *methylation of oligonucleotides by DNA methyltransferases. **A) Duplex 30-mer and 122-mer DNA substrates with hemimethylated top strand (HMTop), hemimethylated bottom strand (HMBot), both strands methylated (DM), and fully unmethylated (UM) were used in the in vitro ^3^H-incorporation assay (counts per minute, CPM) to test the activity of purified Dnmt1. B) Duplex 30-mer and 122-mer DNA substrates with hemimethylated top strand (HMTop), hemimethylated bottom strand (HMBot), both strands methylated (DM), and fully unmethylated (UM) were used in the in vitro ^3^H-incorporation assay (counts per minute, CPM) to test the activity of purified Dnmt3a. C) The single-stranded 122-mer DNA substrates, unmethylated top strand (SSTop), unmethylated bottom strand (SSBot), methylated top strand (SMTop), and methylated bottom strand (SMBot), were used to assay Dnmt1 activity in vitro. D) Single-stranded 122-mer DNA substrates, unmethylated top strand (SSTop), unmethylated bottom strand (SSBot), methylated top strand (SMTop), and methylated bottom strand (SMBot), were used to assay Dnmt3a activity in vitro.

The activity of murine Dnmt3a purified from human cells overexpressing this enzyme was tested in an in vitro methylation assay with in vitro ^3^H-incorporation using fully unmethylated, hemimethylated, and fully methylated 30 bp or 122 bp oligonucleotide substrates. All reactions were carried out in duplicate with no protein and no DNA controls, and all experiments were done multiple times. The no protein controls routinely showed a very low level of ^3^H counts; therefore, the level of radioactivity detected in the no DNA control was treated as background and subtracted from reactions with DNA substrates. Dnmt1 was used as a control in similar reactions. Consistent with previous reports [12,24], Dnmt1 is strongly stimulated by hemimethylated substrates showing approximately a 13-fold higher activity on these substrates than on fully unmethylated DNA and showed nearly no activity on fully methylated DNA (Fig. 1A). Also consistent with the lack of in vitro site preference reported previously [14], virtually no difference in Dnmt1 activity between the 30 bp and the 122 bp substrates was detected (Fig. 1A). This validates the assay and the substrates used.

Consistent with previous reports [12,13], Dnmt3a showed a very low activity on all of the 30 bp substrates, though the highest activity was detected with fully unmethylated DNA (UM); and nearly no activity was detected with fully methylated substrate (DM) (Fig. 1B). A Dnmt3a catalytic mutant [15] showed background levels of radioactivity, confirming the activity of Dnmt3a (data not shown). In contrast, Dnmt3a showed a 20-fold increase in activity on unmethylated 122 bp substrates over the unmethylated 30 bp substrate (UM, Fig. 1B). While near background activity was detected on fully methylated substrates (DM), Dnmt3a displayed the highest activity on fully unmethylated duplex DNA (UM), and its activity on hemimethylated substrates (HMTop and HMBot) was two- to three-fold lower (Fig. 1B). The activity of Dnmt3a on hemimethylated and unmethylated 122 bp substrates is comparable when the number of available unmethylated CpG sites is taken into account. The 30 bp and the 122 bp oligonucleotide substrates have 3 and 4 CpG sites on each strand, respectively. Therefore, the difference in Dnmt3a activity is not proportional to the number of available CpG sites on the 30 bp and the 122 bp substrates. Consistent with our previous findings [15] and results using several other substrates (data not shown), these findings suggest that Dnmt3a has substrate preferences while Dnmt1 does not.

A de novo methylation activity of Dnmt1 has been described as acting on single-stranded substrates at low efficiency in vitro [1,17]. We were interested in determining whether Dnmt3a, with its primary function as a de novo methyltransferase, can also use single-stranded DNA as a substrate. In vitro methylation assays were carried out using unmethylated and methylated single-stranded oligonucleotides as substrates for the analysis of Dnmt1 and Dnmt3a. Dnmt1 showed moderate activity on unmethylated single-stranded DNA (Fig. 1C; SSTop and SSBot). This activity is five-fold lower than its activity on hemimethylated double-stranded DNA substrates (compare with Fig. 1A; HMTop and HMBot) and is two-fold higher than its activity on fully unmethylated duplex DNA (compare with Fig. 1A; UM). These findings are consistent with previous reports [1,18]. Unlike Dnmt1, Dnmt3a showed very little activity on unmethylated single-stranded DNA substrates (Fig. 1D; SSTop and SSBot) and this activity is ten-fold lower than its activity on fully unmethylated double-stranded DNA (compare with Fig. 1B; UM). Both enzymes showed near background activity on methylated single-stranded DNA substrates (SMTop and SMBot in Fig. 1C and Fig. 1D). These findings clearly indicate that Dnmt1 has a stronger de novo methylation activity on single-stranded DNA substrates than on unmethylated duplex DNA, and the de novo methylation activity of Dnmt3a primarily targets duplex DNA substrates with unmethylated CpG sites and not single-stranded DNA. It is also clear that the de novo methylation activity of these two enzymes is quite different in vitro.

### Pre-existing cytosine methylation on the plasmid does not stimulate methylation activity of Dnmt3a in vitro

**Figure 2:**
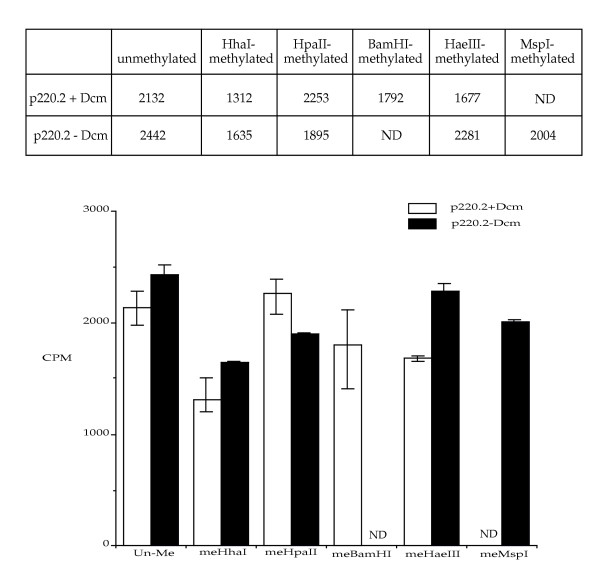
**Pre-existing cytosine methylation does not stimulate Dnmt3a in vitro. **Plasmid DNA extracted from a Dcm-negative *E. coli *strain and a Dcm-positive *E. coli *strain was used in the in vitro ^3^H-incorporation methylation assay (CPM). The average Dnmt3a activities on each plasmid are summarized in the table above the histogram. Both Dcm+ and Dcm-DNA with no CpG methylation (Un-Me), CpG methylation at 32 HhaI sites (meHhaI) or 40 HpaII sites (meHpaII), and non-CpG methylation at 51 HaeIII sites (meHaeIII) were tested. Dcm+ DNA with non-CpG methylation at a single BamHI site (meBamHI) and Dcm- DNA with non-CpG methylation at 40 MspI sites (meMspI) were also tested. ND represents data point not determined.

Previous work indicated that partially-purified human and mouse Dnmt1 has a higher level of methylation activity on partially-methylated plasmids than fully unmethylated plasmids in vitro [19]. Dnmt3a overproduced in *E. coli *showed no increased activity in vitro when three HpaII sites were methylated on a 310 bp substrate, while Dnmt1 showed a 2.9-fold increase in activity, even though the activity reported was low for Dnmt3a [24]. Another report indicated that hemimethylated CCG can stimulate Dnmt3a but not Dnmt1; fully methylated CCG fails to stimulate either enzyme; and fully methylated CWG can stimulate both enzymes [25]. We were interested in further testing whether murine Dnmt3a is stimulated by pre-existing cytosine methylation on the plasmid in vitro. Plasmid DNA is often prepared using Dcm-positive strains of *E. coli*; therefore, it carries methylated cytosines at the internal C of CC(A/T)GG site. For this experiment, plasmid p220.2 DNA was prepared from a Dcm-negative strain (the plasmid DNA thus derived is designated p220.2-Dcm) and from a Dcm-positive strain (plasmid designated p220.2+Dcm) for comparison. The p220.0-Dcm DNA was further methylated in vitro using HhaI (G**C**GC, bold indicates methylation site) methylase, HpaII (C**C**GG) methylase, HaeIII (GG**C**C) methylase, and MspI (**C**CGG) methylase individually. The p220.2+Dcm DNA was also further methylated in vitro using HhaI methylase, HpaII methylase, BamHI methylase (GGAT**C**C), and HaeIII methylase individually. BamHI, HaeIII, and MspI methylases methylate cytosines at non-CpG sites, while HhaI and HpaII methylases methylate cytosines at CpG sites. These in vitro methylated substrates were used in the in vitro methylation assay of Dnmt3a with p220.2+Dcm and p220.0-Dcm DNA as controls. There is very little difference in the Dnmt3a methylation activity between plasmid DNA with or without Dcm methylation, indicating the lack of stimulation by fully methylated CWG sites. Although there are minor variations detected for substrates methylated with different methylases, no stimulation by pre-existing cytosine methylation was observed (Fig. 2). These findings indicate that Dnmt3a is not stimulated by pre-existing symmetrical cytosine methylation at CpG sites or at non-CpG sites on the plasmid substrates in vitro.

### Pre-existing CpG methylation on a minichromosome does not lead to increased Dnmt3a methylation at HhaI or HpaII sites in human cells

**Figure 3:**
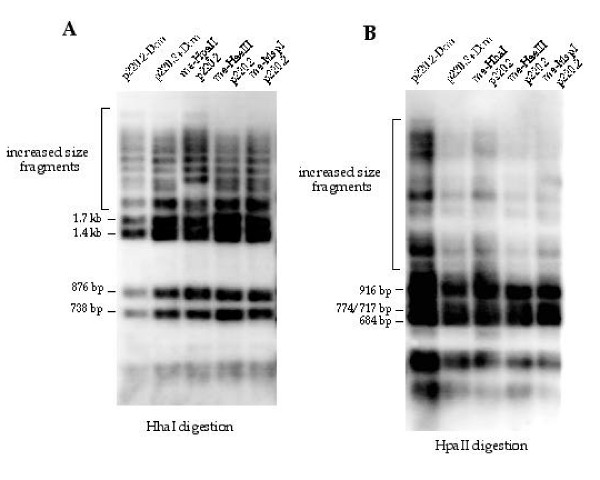
**Southern blot analysis of de novo methylation on plasmid DNA with various types of pre-existing cytosine methylation. **A) Southern hybridization of HhaI-digested plasmid DNA harvested eight days after transfection into human cells. Plasmid DNA with no Dcm methylation (p220.2-Dcm) and with Dcm methylation (p220.2+Dcm) showed no detectable difference in the amount of methylation acquired at HhaI sites. When p220.2-Dcm DNA is methylated in vitro with HpaII methylase (me-HpaII p220.2), HaeIII methylase (me-HaeIII p220.2), or MspI methylase (me-MspI p220.2), and then used in the transfection experiment, no detectable difference due to either p220.2+Dcm or p220.2-Dcm was observed. B) Southern hybridization of HpaII-digested plasmid DNA harvested eight days after transfection into human cells. No detectable difference was observed between p220.2-Dcm, p220.2+Dcm, me-HaeIII p220.2, me-MspI p220.2 and plasmid in vitro methylated with HhaI methylase (me-HhaI p220.2).

To test whether pre-existing CpG methylation can stimulate Dnmt3a activity in vivo, a previously established assay system was used [22]. It has been described previously [22], cotransfection of Dnmt3a expression vector with a target plasmid, such as p220.2, into 293/EBNA1 cells leads to methylation of the target plasmid while no methylation on the target plasmid was detected when the target plasmid was transfected alone or with a Dnmt3a catalytic mutant expression vector into 293/EBNA1 cells. When the transfected plasmid DNA is digested with a methylation-sensitive restriction enzyme, such as HhaI or HpaII, the fraction of the increased size fragments can be compared to the fully digestable fragments in the same lane to assess the activity of Dnmt3a on the plasmid. Any loading variation between lanes does not interfere with this assessment because no comparisons of fragments across lanes are made. p220.2-Dcm plasmid DNA was methylated with HhaI methylase, HpaII methylase, HaeIII methylase, and MspI methylase in vitro before being co-transfected with the Dnmt3a expression vector into 293/EBNA1 cells. The unmethylated p220.2-Dcm and p220.2+Dcm were used as controls in this experiment. Seven days after transfection, the plasmid DNA was harvested using the Hirt method and analyzed by Southern blotting after restriction digestion. There is no appreciable difference in Dnmt3a methylation activity on plasmids with and without Dcm methylation when the methylation sensitive enzyme HhaI is used to assess DNA methylation on p220.2 (Fig. 3A, lanes 1 and 2). There is also little difference in methylation at HhaI sites between HpaII-methylated, HaeIII-methylated, and MspI-methylated p220.2-Dcm plasmid DNA versus plasmid DNA without in vitro methylation (Fig. 3A, lanes 2, 3, 4, and 5). No apparent differences were detected in Dnmt3a methylation activity at HpaII sites between unmethylated plasmid and HhaI-methylated, HaeIII-methylated, and MspI-methylated plasmid DNA (Fig. 3B). When HhaI, HpaII, BamHI, and HaeIII methylases were used to methylate p220.2+Dcm DNA, similar results were observed in the transfection experiments (data not shown). Similar levels of Dnmt3a protein was detected at two days after transfection by Western blot using the Myc antibody in the experiments where Dnmt3a expression was monitored (data not shown). When the same experiments were carried out using 3a-5 cells that overexpress murine Dnmt3a, similar results were seen (data not shown). These results indicate that pre-existing symmetrical methylation at CpG sites or non-CpG sites does not stimulate Dnmt3a methylation at HhaI or HpaII sites in human cells.

### Pre-existing CpG methylation at HpaII or HhaI sites on the minichromosome does not stimulate Dnmt3a methylation at other CpG sites in human cells

To examine whether methylation at CpG sites other than HhaI or HpaII sites is stimulated by pre-existing cytosine methylation, sodium bisulfite sequencing of three different regions in the EBNA1 gene of the plasmid harvested from transfected human cells was carried out. The unmethylated p220.2, HhaI-methylated p220.2, and HpaII-methylated p220.2 plasmids harvested from transfected cells were regionally sequenced for comparison. Methylation at the HhaI and the HpaII sites was excluded from the calculation of methylation because of the pre-existing methylation at these sites on the HhaI-methylated and HpaII-methylated plasmids.

**Figure 4:**
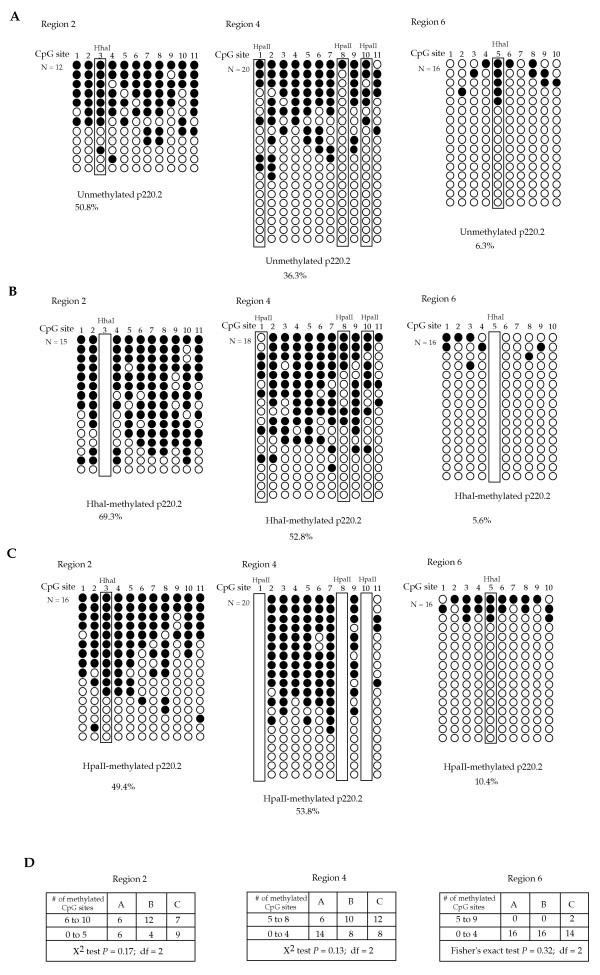
**Sodium bisulfite sequencing of three different regions of the EBNA1 gene from transfected plasmids. **A) Fully unmethylated p220.2, B) HhaI methylated p220.2, and C) HpaII methylated p220.2 were harvested eight days after co-transfection into human cells and sodium bisufite sequenced. Regions 2, 4, and 6 of the EBNA1 gene as designated previously [14] were sequenced. There is a single HhaI site in region 2 and region 6, and there are three HpaII sites in region 4 as indicated on the top of the CpG sites and designated with a box. The percentage of methylation was calculated by dividing the total number of methylation sites detected (excluding methylation found in the HhaI and HpaII sites) by the total number of CpG sites examined (also excluding the HhaI and the HpaII sites). The white rectangular box indicates the premethylated HhaI and HpaII sites on the plasmids. D) Sorting and statistical analysis of the two groups of sequenced molecules from each region. The degrees of freedom (df) are equal to 2. Column A represents molecules from unmethylated p220.2, column B represents molecules from HhaI-methylated p220.2, and column C represents molecules from HpaII-methylated p220.2.

Unmethylated p220.2 showed 50.8% methylation in the EBNA1 region 2, 36.3% methylation in the EBNA1 region 4, and 6.3% methylation in the EBNA1 region 6 (Fig. 4A). HhaI-methylated plasmid p220.2 had 69.3%, 52.8%, and 5.6% of methylation in EBNA1 regions 2, 4, and 6, respectively (Fig. 4B). Similar levels of methylation (49.4%, 53.8%, and 10.4% in EBNA1 regions 2, 4, and 6, respectively) were found on HpaII-methylated p220.2 (Fig. 4C). The patterns of methylation in all three regions of the EBNA1 gene were also similar for these three plasmids (Fig. 4). Previously, 55.3 % and 10.6% of methylation were detected in the EBNA1 region 2 and region 6, respectively, in similar experiments [15]. The percentage of overall methylation is higher in region 2 of HhaI-methylated p220.2 than unmethylated and HpaII-methylated p220.2. The percentage of overall methylation in region 4 is also higher in both HhaI-methylated and HpaII-methylated p220.2 than unmethylated p220.2. However, the patterns of methylation are similar for all plasmids in all three regions. We reasoned that if preexisting methylation HhaI or HpaII sites can stimulate Dnmt3a in vivo, a larger fraction of highly methylated molecules should be observed in the transfected HhaI-methylated and HpaII-methylated p220.2 plasmid when compared with unmethylated p220.2. Therefore, the sequenced molecules were sorted into two groups, a group with more than 50% of the CpG sites methylated and one with less than 50% of the CpG sites methylated. A chi-square test was then carried out to test the hypothesis that there is no significant difference between the number of molecules in these two groups from p220.2, HhaI-methylateed-p220.2, and HpaII-methylated p220.2 plasmids. Fisher's exact test was used to test the same hypothesis for results from region 6 because no molecules from unmethylated and HhaI-methylated p220.2 had more than 50% of the CpG sites methylated. The *P *values of the statistical tests were 0.17, 0.13, and 0.32 for regions 2, 4, and, 6, respectively (Fig. 4D), indicating that the difference is not significant. The same tests were carried out for regions 2 and 4 with a different cut-off for the sorting to ensure that this does not change the outcome. When the sequenced molecules from region 2 were sorted as one group with 5 to 10 methylated CpG sites and another group with 0 to 4 methylated CpG sites, the *P *value of the chi-square test is 0.37. When the sequenced molecules from region 4 were sorted into a group with 4 to 8 methylated CpG sites and a group with 0 to 3 methylated sites, the *P *value of the chi-square test is 0.22. Therefore, the higher level of methylation observed in the EBNA1 regions 2 and 4 on HhaI-methylated plasmid and in the EBNA1 regions 2 and 6 on HpaII-methylated plasmid is considered within normal experimental variation and not due to stimulation of Dnmt3a by the pre-existing methylation at HhaI of HpaII sites. These findings indicate that there is no stimulation of methylation at other CpG sites in human cells when HhaI or HpaII sites carry pre-existing cytosine methylation. It is noteworthy that no methylation at non-CpG sites was detected in all three regions sequenced.

## Discussion

It has been described that the de novo methylation activity of Dnmt1 can act on single-stranded DNA [1,17]. This leads to the hypothesis that single-stranded DNA formed during replication or repair might serve as a nucleation site for de novo methylation [17]. Several studies suggest that CpG islands may serve as DNA replication origins [for a review see [26]]. As first described by Baylin in 1986 [27], hypermethylation of CpG islands has been well recognized to occur in cancer. It is possible that the single-stranded DNA methylation activity of Dnmt1 targets it to CpG islands that are DNA replication origins and initiates de novo methylation in the genome. Since the primary function of Dnmt3a is de novo methylation, it is important to know whether Dnmt3a has such an activity. Unlike Dnmt1, we find here that Dnmt3a does not act on single-stranded oligonucleotides in vitro. Although one previous study [28] reported Dnmt3a methylation of single-stranded oligonucleotide substrates, the conclusion remains unclear based on the substrate used in that study. The substrate used in Yokochi and Robertson [28] has 10 CpG sites on an oligomer which is 34 nucleotides in length and which has GATC on both ends; therefore, it can form double-stranded regions over substantial lengths both intra- and inter-molecularly under low salt and moderately high temperature conditions. In contrast, the 122 bases oligonucleotide substrate used in this study does not form CpG pairing either intra- or inter-molecularly based on several oligonucleotide analysis software packages. Furthermore, no other conformation was observed when the radioactive-labeled oligonucleotide was resolved on a native gel either as single-stranded or double-stranded configuration (data not shown).

In our study, Dnmt3a showed a much higher activity on the 122 bp double-stranded DNA substrate than on the 30 bp substrate, indicating some form of substrate preference. We have tested other substrates ranging from 83 bp to 130 bp and the substrate length or the potential processivity of the enzyme is not the basis for these differences (data not shown), indicating that local DNA sequence is the basis. These findings suggest functional differences for the de novo methylation activity of these two enzymes. Based on our study here, if de novo methylation occurs at CpG sites within DNA with single-stranded features in vivo, then Dnmt3a is not likely to be the methyltransferase responsible for this process. Also, Dnmt3a may prefer certain sequences while Dnmt1 does not discriminate among its targets.

It has been shown that pre-existing cytosine methylation can stimulate de novo methylation activity of Dnmt1 in vitro both in cis and in trans [17-19]. This suggests that Dnmt1 can be stimulated by hemimethylated DNA as well as symmetrically methylated DNA. In a previous study, Dnmt3a overproduced in *E. coli *showed no increased activity when three HpaII sites were methylated on a 310 bp substrate, even though the activity of Dnmt3a was low [24]. However, another study showed stimulation of Dnmt3a by hemimethylated CCG and fully methylated CWG [25]. Here, we performed both in vitro and in vivo testing of whether pre-existing cytosine methylation can stimulate Dnmt3a, including fully methylated CCG and CWG sites. We find that Dcm+ plasmid DNA does not stimulate Dnmt3a activity in vitro when compared with plasmid DNA without methylation at these Dcm sites, suggesting the lack of stimulation by methylation at CWG sites. This finding is at odds with the finding of Kim et al. [25]. There are several non-CpG sites on the methylated CWG and CCG substrates used in Kim et al. [25]. It is possible that CWG methylation does not stimulate CpG methylation but stimulates a non-CpG methylation activity in those reactions, since the Dnmt3a used in Kim et al. was demonstrated to have non-CpG methylation activity [25]. The Dnmt3a used in our study has no detectable non-CpG methylation activity; therefore, no increased activity was observed with pre-existing cytosine methylation. However, it is intriguing that Dnmt3a is not stimulated by hemimethylated CpG, fully methylated CpG, or fully methylated CCG, and yet it is stimulated by hemimethylated CCG and fully methylated CWG in vitro [25]. Whether this difference is due to the expression of the enzyme in insect cells versus human cells would need further investigation. There is also no stimulation of the Dnmt3a activity in vitro by cytosine methylation at other CpG or non-CpG sites on the plasmid with or without Dcm methylation. These findings indicate a clear difference between the de novo methylation activity of Dnmt1 and Dnmt3a in vitro.

Transfection experiments also showed that pre-existing cytosine methylation at CpG sites or non-CpG sites does not stimulate Dnmt3a to methylate CpGs at HhaI and HpaII sites on the minichromosome. Sodium bisulfite sequencing of the transfected minichromosome further showed that methylation at HhaI or HpaII sites does not lead to increased methylation at other CpG sites. Methylation at non-CpG sites by Dnmt3a was not detected in any of the molecules sequenced, indicating the lack of non-CpG methylation by Dnmt3a in this particular human cell line. These findings demonstrate that pre-existing cytosine methylation does not stimulate Dnmt3a activity for either CpG or non-CpG methylation in human cells.

It has been proposed that Dnmt1 plays a role in DNA methylation spreading in mammalian cells [24]. In our previous transfection experiments using patch-methylated minichromosomes, no spreading of methylation from the methylated patch was observed [23]. We also have not observed de novo methylation on the transfected minichromosomes with methylation at HhaI or HpaII sites by bisulfite sequencing (data not shown). These findings indicate the lack of de novo methylation activity on these minichromosomes without overexpression of Dnmt3a in human cells. Although it remains unclear which methyltransferases are involved in methylation spreading, the fact that Dnmt3a is not stimulated by hemimethylated DNA or by symmetrically methylated DNA strongly suggests that Dnmt3a does not participate in this process. Consistent with the findings in Fatemi et al [24], we have found that plasmid DNA premethylated with Dnmt3a stimulates Dnmt1 activity more than two-fold in vitro (data not shown). In our previous study, Dnmt3a generates an asymmetrical methylation pattern on the two DNA strands in vitro [15].

**Figure 5:**
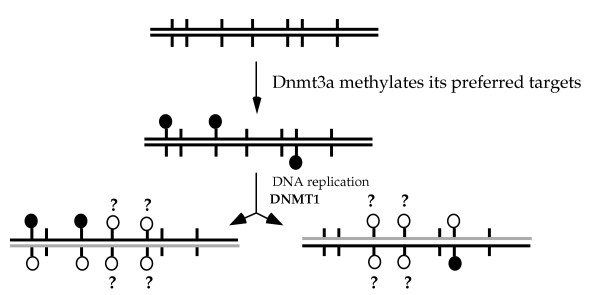
**Model for one of the de novo methylation processes carried out by Dnmt3a and Dnmt1. **Dnmt3a initiates methylation on one DNA strand at its preferred CpG sites (filled circles). After DNA replication, Dnmt1 methylates the other strand (open circles) at these hemimethylated sites. The hemimethylated DNA may also stimulate Dnmt1 for further methylation at previous unmethylated CpG sites (open circles indicated with ?).

Therefore, it is very likely that one of the processes for de novo methylation is for Dnmt3a to initiate methylation on one DNA strand; then this hemimethylated DNA would lead to the stimulation of Dnmt1 for further methylation (Fig. 5). This activity of Dnmt1 would be in addition to its known methylation of hemimethylated sites after DNA replication (Fig. 5).

## Conclusion

In this study, we demonstrated several novel differences between Dnmt1 and Dnmt3a de novo methylation activity. Unlike Dnmt1, Dnmt3a produced in human cells has very little activity on single-stranded DNA in vitro. While Dnmt1 shows higher de novo methylation activity on single-stranded substrates than on unmethylated duplex DNA, Dnmt3a primarily acts on unmethylated sites on duplex DNA. Dnmt1 shows no preference for substrates with different sequence or length, whereas Dnmt3a shows preference for different substrates. In contrast to Dnmt1, Dnmt3a is not stimulated by pre-existing cytosine methylation on plasmids either at CpG sites or non-CpG sites in vitro. This is the first study demonstrating that pre-existing cytosine methylation does not stimulate the activity of Dnmt3a at CpG or non-CpG sites in human cells. The de novo methylation activity of these two enzymes is clearly different.

## Methods

### Oligonucleotide substrates

The 30-mer double-stranded oligonucleotide substrates used in this study are identical to the ones used in Pardhan et al [1] and Okano et al [12] with the addition of one hemimethylated substrate with the CpG methylation on the anti-parallel DNA strand. The 30-mer oligonucleotides were synthesized by Operon. The top strand of the 122-mer substrate, 5'-GATACATATTTGAATGTATTTAGAAAAATAAACAAATAGGGGTTC CGCGCACATTTCCCCGAAAAGTGCCACCTGACGTCTAAGAAACCATTATT ATCATGACATTAACCTATAAAAATAGG-3', was derived from the EBNA1 sequence that contains 4 CpG sites. The bottom strand of the substrate is the complementary sequence of the top strand. The 122-mer oligonucleotides were synthesized and purified by the Microchemical Core facility of the Norris Cancer Center. The double-stranded substrates were generated by adding equimolar amounts of the complementary strands in 10 mM TrisHCl (pH 8.0) and 100 mM NaCl, boiling for 10 min, and slowly cooling in a bath of 300 ml water at room temperature. The annealing of the oligonucleotides was checked by PAGE. The hemimethylated HMTop substrates were generated using a top strand oligonucleotide with methyl cytosines at all the CpG sites and a fully unmethylated bottom strand oligonucleotide. The hemimethylated HMBot substrates were made with unmethylated top strand and fully methylated bottom strand oligonucleotides. The fully methylated substrates (DM) contain methylated top and methylated bottom strand oligonucleotides. The fully unmethylated substrates (UM) were generated with unmethylated top and bottom strand oligonucleotides.

### Enzymes and in vitro ^3^H-incorporation assay

Human Dnmt1 was purchased from New England Biolabs. The GST-Dnmt3a (GST-3a) and GST-Dnmt3a mutant (GST-3aMut) fusion proteins were expressed in 293T cells and purified using glutathione-agarose beads as described previously [15]. In vitro methylation activity of Dnmt1, Dnmt3a, and Dnmt3a mutant was measured by ^3^H-incorporation assay. In vitro methylation was carried out for Dnmt3a and the Dnmt3a mutant in a 20 ul reaction with 314 fmol of enzyme in 10 mM TrisHCl (pH 8.0), 1 mM EDTA, 1 mM DTT, and 1.1 uM ^3^H-AdoMet (New England Nuclear, 14.7 Ci/mmol) at 37°C overnight (16 hours). The same assay was carried out for Dnmt1 (New England Biolabs) in a 25 ul reaction with 1.1 uM ^3^H-AdoMet in the manufacturer's recommended buffer using 2.5 units of the enzyme. In each reaction, 0.25 pmol of the duplex substrate or 0.5 pmol of the single-stranded substrate was used. The in vitro methylation reaction was treated with Proteinase K at 55°C for 1 h before being spotted onto the DE-81 filters and washed as described previously [29]. The radioactivity retained on the air-dried DE-81 filters was measured by scintillation counting (Packard Tri Carb 2100TR) with 2 ml of scintillation fluid.

### Plasmid and in vitro methylation of plasmid

The plasmid p220.2 [30] was used throughout the study. Plasmid p220.2 DNA without Dcm methylation, designated p220.2-Dcm, was prepared from a Dcm-negative strain, GM127. Plasmid DNA with Dcm methylation, designated p220.2+Dcm, was prepared from a Dcm-positive strain, DH10B. The p220.0-Dcm DNA was further methylated in vitro using HhaI methylase, HpaII methylase, HaeIII methylase, and MspI methylase individually. The p220.2+Dcm DNA was further methylated in vitro using prokaryotic methylase, HhaI methylase, HpaII methylase, BamHI methylase, or HaeIII methylase individually. MeHhaI-p220.2 was in vitro methylated at all HhaI sites using the HhaI-methylase (New England Biolabs) under the conditions recommended by the manufacturer. MeHpaII-p220.2 was methylated at HpaII sites according to the conditions suggested by the manufacturer using the HpaII methylase (New England Biolabs). Two cytosine methylases that methylate non-CpG sites, BamHI methylase and HaeIII methylase, were used to generate meBamHI-p220.2 and meHaeIII-p220.2, respectively. There are 32 HhaI sites, 40 HpaII sites, a single BamHI site, 51 HaeIII sites, and 40 MspI sites on p220.2. After methylation, DNA was extracted with phenol-chloroform followed by ethanol precipitation. The methylation status was verified by restriction enzyme digestion using the appropriate enzyme for the specific methylase used.

### Cell lines and transfection

The 293/EBNA1 and 3a-5 cells were used in this study. The 3a-5 cells were derived by integrating a Dnmt3a expression construct into the 293/EBNA1 cells [22]. Throughout the study, the calcium phosphate transfection method was used to introduce DNA into the human cell line. In some experiments, wildtype or mutant Dnmt3a expression vector [22], pMT3aMyc and pMT3aMut, was cotransfected with the assay plasmid into 293/EBNA1 cells.

### DNA recovery and analysis

Plasmid DNA was harvested from the cells by the Hirt method [31] when the cells reached confluence after transfection. A small fraction of the cells was re-seeded for later harvests. The plasmid DNA harvested from the transfected cells was digested with HhaI or HpaII enzyme, fractionated on 1% agarose gel, and analyzed by Southern blot analysis using the entire plasmid as a probe.

### Bisulfite genomic sequencing and statistical analysis

Bisulfite genomic sequencing was carried out as described previously [32] with minor modifications. Three different regions, regions 2, 4, and 6, of the EBNA1 gene, on the plasmid p220.2 were amplified and ligated into the TOPO TA cloning vector (Invitrogen). The locations of these three regions as well as primer sequences and PCR conditions have been described previously [14]. Both strands of each clone were sequenced using the EXCEL II sequencing kit (Epicentre) and analyzed on the Li-Cor 4200 sequencer. Bisulfite sequencing results for each of the three regions from p220.2, HhaI-methylated p220.2, and HpaII-methylated p220.2 were compared. Molecules were sorted into two groups: one with more than 50% of the CpG sites methylated and one with less than 50% of the CpG sites methylated. A chi-square test was carried out to test the hypothesis that there is no significant difference between the number of molecules in each of the two groups observed from p220.2, HhaI-methylateed-p220.2, and HpaII-methylated p220.2 plasmids. A Fisher's exact test was used to test the same hypothesis for results from region 6 because nearly all of the molecules had less than 50% of the CpG sites methylated.
